# Rosiglitazone Reverses Inflammation in Epididymal White Adipose Tissue in Hormone-Sensitive Lipase-Knockout Mice

**DOI:** 10.1016/j.jlr.2022.100305

**Published:** 2022-10-20

**Authors:** Petra Kotzbeck, Ulrike Taschler, Christoph Haudum, Ines Foessl, Gabriele Schoiswohl, Beate Boulgaropoulos, Kaddour Bounab, Johanna Einsiedler, Laura Pajed, Anna Tilp, Anna Schwarz, Thomas O. Eichmann, Barbara Obermayer-Pietsch, Antonio Giordano, Saverio Cinti, Rudolf Zechner, Thomas R. Pieber

**Affiliations:** 1 Division of Endocrinology and Diabetology, Medical University of Graz, Graz, Austria; 2 Institute of Molecular Biosciences, University of Graz, Graz, Austria; 3 Research Unit for Tissue Regeneration, Repair and Reconstruction, Division of Plastic, Aesthetic and Reconstructive Surgery, Department of Surgery, Medical University of Graz, Graz, Austria; 4 Cooperative Centre for Regenerative Medicine (COREMED), Joanneum Research Forschungsgesellschaft m.b.H, Graz, Austria; 5 Department of Pharmacology and Toxicology, University of Graz, Graz, Austria; 6 Institute for Biomedicine and Health Sciences (HEALTH), Joanneum Research Forschungsgesellschaft m.b.H, Graz, Austria; 7 Center for Explorative Lipidomics, BioTechMed-Graz, Graz, Austria; 8 Department of Experimental and Clinical Medicine, Center of Obesity, University of Ancona (Politecnica delle Marche), Ancona, Italy; 9 BioTechMed-Graz, Graz, Austria

**Keywords:** adipocytes, adipose tissue, lipolysis, FA metabolism, lipase, inflammation, dysfunctional adipocytes, FA, electron microscopy, lipotoxicity, ATGL, adipose triglyceride lipase, cDNA, complementary DNA, Cer, ceramide, CLS, crown-like structure, DAG, diacylglycerol, ER, endoplasmic reticulum, eWAT, epididymal white adipose tissue, HSL, hormone-sensitive lipase, iWAT, inguinal white adipose tissue, TAG, triacylglycerol, WAT, white adipose tissue

## Abstract

Hormone-sensitive lipase (HSL) plays a crucial role in intracellular lipolysis, and loss of HSL leads to diacylglycerol (DAG) accumulation, reduced FA mobilization, and impaired PPARγ signaling. *Hsl* knockout mice exhibit adipose tissue inflammation, but the underlying mechanisms are still not clear. Here, we investigated if and to what extent HSL loss contributes to endoplasmic reticulum (ER) stress and adipose tissue inflammation in *Hsl* knockout mice. Furthermore, we were interested in how impaired PPARγ signaling affects the development of inflammation in epididymal white adipose tissue (eWAT) and inguinal white adipose tissue (iWAT) of *Hsl* knockout mice and if DAG and ceramide accumulation contribute to adipose tissue inflammation and ER stress. Ultrastructural analysis showed a markedly dilated ER in both eWAT and iWAT upon loss of HSL. In addition, *Hsl* knockout mice exhibited macrophage infiltration and increased *F4/80* mRNA expression, a marker of macrophage activation, in eWAT, but not in iWAT. We show that treatment with rosiglitazone, a PPARγ agonist, attenuated macrophage infiltration and ameliorated inflammation of eWAT, but expression of ER stress markers remained unchanged, as did DAG and ceramide levels in eWAT. Taken together, we show that HSL loss promoted ER stress in both eWAT and iWAT of *Hsl* knockout mice, but inflammation and macrophage infiltration occurred mainly in eWAT. Also, PPARγ activation reversed inflammation but not ER stress and DAG accumulation. These data indicate that neither reduction of DAG levels nor ER stress contribute to the reversal of eWAT inflammation in *Hsl* knockout mice.

Hormone-sensitive lipase (HSL) catalyzes the release of FAs from adipose tissue depots in times of energy demand. Upon hormonal stimulation, lipolysis is activated, and the enzymes adipose triglyceride lipase (ATGL), HSL, and monoacylglycerol lipase successively hydrolyze triacylglycerol (TAG) (1). HSL has the highest affinity for diacylglycerol (DAG) (2, 3, 4), and *Hsl* knockout in mice provokes DAG accumulation in various tissues (e.g., white adipose tissue [WAT] and brown adipose tissue, testes, and brain (3)), reduced FA mobilization, and impaired PPARγ signaling (5, 6). HSL loss also promotes adipocyte hypertrophy in WAT and leads to decreased overall WAT mass, which occurs because of impaired PPARγ signaling in *Hsl* knockout mice. Upon aging, *Hsl* knockout mice show a significant decrease in visceral and subcutaneous WAT mass (3, 6, 7). Pharmacological activation of PPARγ with the PPARγ agonist rosiglitazone was shown to counteract WAT loss in *Hsl* knockout mice (6). In contrast to *Hsl* knockout mice, which show little effect on glucose metabolism, humans with *HSL* mutations are prone to develop type 2 diabetes, hyperlipidemia, and hepatic steatosis (8). Results from an Old Amish Order, whose participants were homozygous for a loss-of-function *HSL* mutation, have revealed modestly reduced fat content in lower extremities similar to *Hsl* knockout mouse studies. Fat biopsies in the Amish population showed increased mRNA expression of inflammatory marker genes and reduced ATGL protein abundance (8).

In both mice and humans, increase of visceral WAT mass is linked to insulin resistance and type 2 diabetes, whereas an increase of subcutaneous WAT mass is considered to be healthier (9). Especially visceral WAT of *Hsl* knockout mice has shown signs of inflammation and adipocyte death (10, 11). Although inflammation and hypertrophy of visceral WAT is predictive for the development of obesity-associated complications, *Hsl* knockout mice remain insulin sensitive and are even protected against diet-induced obesity (12, 13, 14). In obese humans, *HSL* expression is decreased, whereas basal lipolysis is markedly increased (15, 16). Decreased HSL activity causes DAG accumulation in *Hsl* knockout mice (3) and human tissue (8). DAG and ceramide (Cer) accumulation has been associated with the development of endoplasmic reticulum (ER) stress, cell death, inflammation, and insulin resistance (17, 18, 19, 20, 21, 22, 23, 24, 25). However, the association of HSL loss and DAG or Cer accumulation to ER stress development and inflammation in WAT still needs further clarification.

Although HSL loss has been extensively studied in mice and humans, the effects of HSL loss on adipose tissue dysfunction and inflammation and also to what extent epididymal WAT (eWAT) and inguinal WAT (iWAT) are affected remain unclear. Furthermore, it is not known yet whether DAG accumulation in WAT of *Hsl* knockout mice induces ER stress and if it is a cause or consequence of adipose tissue inflammation.

The aim of this study was to investigate, if and to what extent HSL loss contributes to ER stress and WAT inflammation in eWAT and iWAT of *Hsl* knockout mice. Furthermore, we aimed to investigate how impaired PPARγ signaling affects the development of inflammation in those adipose tissue depots. In addition, we wanted to study to what extent DAG and Cer accumulation contributes to adipose tissue inflammation and ER stress.

We analyzed ER stress, PPARγ signaling, and inflammation in eWAT and iWAT of adult *Hsl* knockout mice under basal conditions and after chronic rosiglitazone treatment. DAG and Cer levels in eWAT were also analyzed to elucidate the contribution of DAG and Cer accumulation to adipose tissue inflammation and potential ER stress development.

## Materials and Methods

### Animals

*Hsl* knockout mice were generated by targeted homologous recombination as described previously (3) and backcrossed at least 10 times to a C57Bl/6J genetic background. Mice were kept on a standard laboratory chow diet containing 4.5% (w/w) fat (sniff Spezialdiäten GmbH, Soest, Germany). For all studies, male mice with an age range of 10–14 weeks were used. Animals had ad libitum access to food and water and were housed with a light and dark period of 10 and 14 h, respectively, at a room temperature of 22°C ± 1°C. For refeeding conditions, mice were fasted for 12 h and had then free access to food for 2 h. Body mass composition was assessed in nonanesthetized mice using the time-domain NMR minispec (Live Mice Analyzer system, Model LF90II; Bruker Optik, Germany). Animals were anesthetized with ISOflo®/isoflurane (Abbott, Abbott Park, IL) and sacrificed by cervical dislocation. All experiments were approved by the Austrian Federal Ministry for Science and the local ethics committee (BMWF-66.007/0017-II/3b/2013).

### Rosiglitazone diet

For PPARγ agonism, animals were fed standard chow diet containing 200 mg rosiglitazone (Caymen Chemical, MI) per kilogram chow diet for 20 days or 28 days. During dietary intervention, body weight was monitored regularly.

### Blood parameters

Blood was collected by retro-orbital puncture of anesthetized mice. Plasma levels of nonesterified FA, glycerol, and TAG were measured using the commercially available kits NEFAC (WAKO Chemicals, Germany), TG Infinity Reagent (Thermo Fisher Scientific, MA), and Free Glycerol Reagent (Merck, Darmstadt, Germany). Plasma leptin was determined using a mouse leptin ELISA (catalog no.: 90030; Crystal Chem, IL).

### Histological analysis

eWAT and iWAT tissues were fixed in 10% neutrally buffered formalin solution, and tissue was processed in a Tissue-Tek VIP (Sakura, Germany). Three 3 μm thick sections were prepared and attached to charged glass slides (Menzel Superfrost Plus; Thermo Fisher Scientific). Antigen retrieval was performed for 20 min at 90°C in a Decloaking Chamber (DC2012; Biocare Medical, CA) in 10 mM sodium citrate buffer (pH 6) with 0.5% Tween-20. Immunohistochemistry was performed with a monoclonal anti-Mac-2 primary antibody (1:500 dilution; catalog no.: CL8942AP, Cedarlane, Canada), the Vectastain ABC HRP rat kit (catalog no.: PK-4004; Vector Labs, CA), Sigmafast DAB for visualization (catalog no.: D4293: Merck), and hematoxylin for counterstaining. Images were captured with an Aperio ScanScope AT (Leica Biosystems, Austria) digital slide scanner at 40-fold magnification. The SlideJ plugin (26) was used in connection with ImageJ (Fiji distribution) to split the Aperio digital slides into TIFF images (27). The Adiposoft plugin was used to detect and count adipocytes (28). Crown-like structures (CLSs) were counted by two persons on two different sections per sample. CLSs were defined by a minimum of three macrophages surrounding an individual adipocyte remnant.

### Transmission electron microscopy

Small fragments of eWAT and iWAT tissue from perfused mice were fixed in 2% glutaraldehyde-2% paraformaldehyde in phosphate buffer for 4 h at room temperature, postfixed in 1% osmium tetroxide, dehydrated in a graded series of acetone, and embedded in an Epon-Araldite mixture. To determine the region of interest, semithin sections were cut and stained with toluidine blue. Thin sections were obtained with an MT-X Ultratome (RMC, Tucson, AZ), stained with lead citrate, and examined with a CM10 transmission electron microscope (Philips; Eindhoven, The Netherlands).

### Tissue homogenization and Western blotting analysis

Snap-frozen tissues were homogenized in ice-cold homogenization buffer (0.25 M sucrose, 1 mM EDTA, 1 mM DTT, pH 7) containing HALT protease and phosphatase inhibitors (Thermo Fisher Scientific) using a hand-held disperser (Ultra-Turrax, IKA, Germany). Samples were centrifuged for 15 min at 1,000 *g* at 4°C. The infranatant was collected, and protein concentration was assayed using the Bio-Rad Protein Assay Dye Reagent (Bio-Rad Laboratories GmbH, Germany). Proteins were separated using SDS-PAGE, and proteins were transferred to a polyvinylidene difluoride membrane (Bio-Rad Laboratories GmbH). Specific proteins were detected using the anti-rabbit peIF2 alpha (Ser51) (1:1,000 dilution, catalog no.: 3398, Cell Signaling Technology, MA) and eIF2 alpha (1:1,000 dilution, catalog no.: 5324, Cell Signaling Technology). Alpha tubulin (1:1,000 dilution, catalog no.: ab52866, Abcam, UK) was used as a loading control. As secondary antibody, HRP-conjugated goat anti-rabbit antibody (Cell Signaling Technology) was used. Proteins were visualized using Clarity™ substrate and the ChemiDoc™system (Bio-Rad Laboratories GmbH, Germany). Signal density was determined with ImageJ (29) or directly the ChemiDoc™system (Bio-Rad Laboratories GmbH).

### RNA isolation and quantitative real-time PCR

Tissue homogenization and RNA isolation of frozen tissues was performed using Qiazol reagent and the RNeasy Mini Kit (both Qiagen Vertriebs GmbH, Austria) according to standard protocols. For gene expression analyses, RNA samples were treated with DNase and reversely transcribed into single-stranded complementary DNA (cDNA) with the iScript™ gDNA clear cDNA synthesis kit (Bio-Rad Laboratories GmbH). cDNA samples were amplified using the SsoAdvanced™ universal SYBR green supermix (Bio-Rad Laboratories GmbH) or the TaqMan™ Gene Expression Master Mix (Thermo Fisher Scientific) and target gene-specific primer pairs (Sigma-Aldrich, MO) or TaqMan™ probes. Primer sequences are available on request. Real-time PCR was run on the C1000 Thermocycler, using the CFX384 Real-Time System (Bio-Rad Laboratories GmbH). Relative target gene expression was normalized to the ribosomal gene *36b4* or hypoxanthine-guanine-phosphoribosyltransferase (*Hprt*) and calculated using the method published by Pfaffl (30).

### Tissue lipid analysis

Lipids of weighed tissue explants were extracted twice with 4 ml chloroform/methanol (2/1, v/v) containing 500 pmol butylated hydroxytoluene, 1% acetic acid, and 150 pmol of internal standards (14:0-14:0 DAG, d18:1/17:0 Cer; Avanti Polar Lipids, AL) per sample according to Folch *et al.* (31). Extraction was performed with continuous shaking for 30 min at room temperature. After addition of 800 μl H_2_O and further incubation for 30 min at room temperature, samples were centrifuged at 1,000 *g* for 15 min at room temperature to establish phase separation. The lower organic phase was collected, 2.5 ml chloroform was added to the remaining aqueous phase, and a second extraction was performed as described above (30 min at room temperature with subsequent centrifugation). Combined organic phases were dried under a stream of nitrogen and resolved in 800 μl of methanol/2-propanol/water (6/3/1, v/v/v) for ultraperformance LC-MS analysis.

Chromatographic separation was modified according to Knittelfelder *et al.* (32) using an ACQUITY-UPLC system (Waters Corporation, UK) equipped with a Kinetex C18 column (2.1 × 50 mm, 1.7 μm; Phenomenex, CA) starting a 15 min linear gradient with 80% solvent A (MeOH/H_2_O, 1/1, v/v; 10 mM ammonium acetate, 0.1% formic acid, and 8 μM phosphoric acid).

An EVOQ Elite™ triple quadrupole mass spectrometer (Bruker, Germany) equipped with an electrospray ionization source was used for detection. DAG and Cer species were analyzed by selected reaction monitoring using (M + NH_4_)^+^ to (RCOO + 58)^+^ of the respective esterified FA as transition (15 eV collision energy, 60 ms, 0.7 resolution for Q1/Q3) for DAG and (M + H)^+^ to *m/z* 264 (22 eV collision energy, 60 ms, 0.7 resolution for Q1/Q3) for Cer. Data acquisition was done by MS Workstation (Bruker). Data were normalized for recovery, extraction efficacy, and ionization efficacy by calculating analyte/internal standard ratios (AU) and expressed as AU/g tissue.

### Statistical analysis

Data are presented as mean + SD. Data were tested for normality, and statistical significance was determined using unpaired two-tailed Student’s *t*-test or two-way ANOVA for multiple comparisons. Multiple testing was corrected by Tukey or Holm-Sidak posthoc test. Graphs and statistics were prepared in GraphPad Prism, version 8.0 (GraphPad Software, Inc). Group differences were considered significant for (∗) *P* < 0.05, (∗∗) *P* < 0.01, and (∗∗∗) *P* < 0.001.

## Results

### HSL loss promotes ER stress in eWAT and iWAT

To investigate the contribution of HSL loss to the development of ER stress in adipocytes and adipose tissue, the expression of the ER stress markers *Bip/Grp78*, Chop, the XBP-1 target genes, *Erdj4* and *Edem*, and phosphorylation of eIF2 alpha were analyzed in eWAT and iWAT of wild-type and *Hsl* knockout mice.

**Fig. 1:**
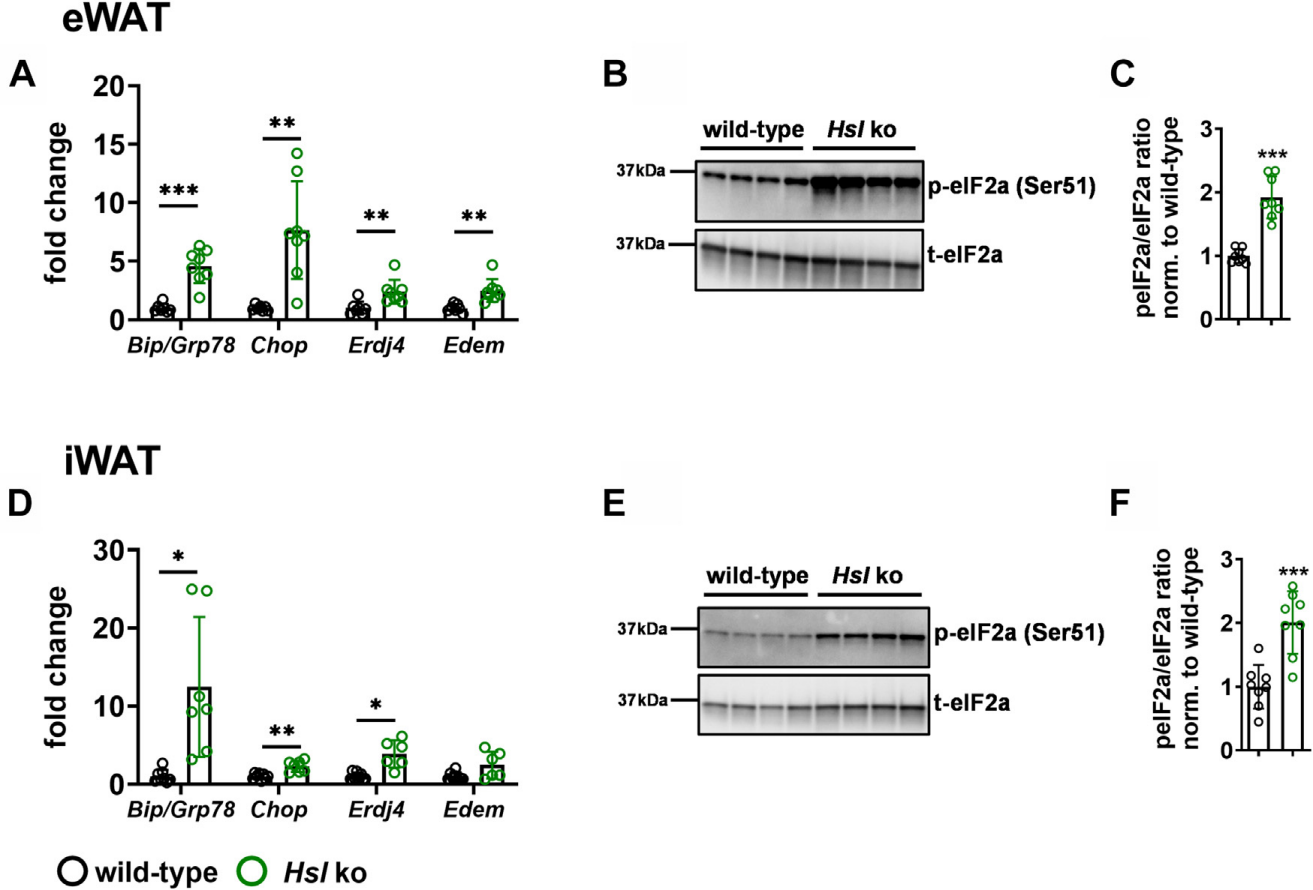
ER stress markers are significantly increased in eWAT and iWAT of *Hsl* knockout mice. A: mRNA expression of XBP1 target genes *Erdj4* and *Edem*, *Bip/Grp78*, and *Chop* in eWAT. Target gene abundance was normalized to *36b4* and hypoxanthine-guanine-phosphoribosyltransferase (*Hprt*) and expressed relative to wild type of each marker (*n* = 7–8). B: Representative immunoblot of phosphorylated eIF2alpha (peIF2a) and total eIF2alpha (teIF2a) of eWAT of *Hsl* knockout mice and wild-type mice. C: Quantification of pEIF2alpha/total peIF2 alpha ratio (*n* = 8). D: mRNA expression of XBP1 target genes *Erdj4* and *Edem*, *Bip/Grp78*, and *Chop* in iWAT. Target gene abundance was normalized to *Hprt* and expressed relative to wild type of each marker (*n* = 6–8). E: Representative immunoblot of phosphorylated eIF2alpha (peIF2a) and total eIF2alpha (teIF2a) of iWAT of wild-type and *Hsl* knockout mice. F: Quantification of pEIF2alpha/total peIF2 alpha ratio (*n* = 8). Data are represented as mean ± SD. Statistical significance was determined using unpaired two-tailed Student’s *t*-test corrected for multiple comparisons using the Holm-Sidak method. (∗) *P* < 0.05; (∗∗) *P* < 0.01; and (∗∗∗) *P* < 0.001.

*Hsl* knockout mice showed a robust increase in ER stress markers in eWAT and iWAT (Fig. 1). XBP-1 target gene expression of *Erdj4* and *Edem* in eWAT was significantly increased in *Hsl* knockout mice compared with wild-type mice (Fig. 1A). Consistent with this finding, *Bip/Grp78* and *Chop* mRNA levels were elevated in eWAT of *Hsl* knockout mice (Fig. 1A). eIF2alpha phosphorylation was significantly increased (1.9-fold) relative to wild-type mice (Fig. 1B, C). Gene expression of the ER stress markers, *Erdj4*, *Bip/Grp78*, and *Chop*, was also significantly increased in iWAT, whereas *Edem* expression only showed a trend toward increased expression (Fig. 1D). In iWAT, eIF2alpha phosphorylation was 2-fold increased, similar to the results obtained in eWAT (Fig. 1E, F).

**Fig. 2:**
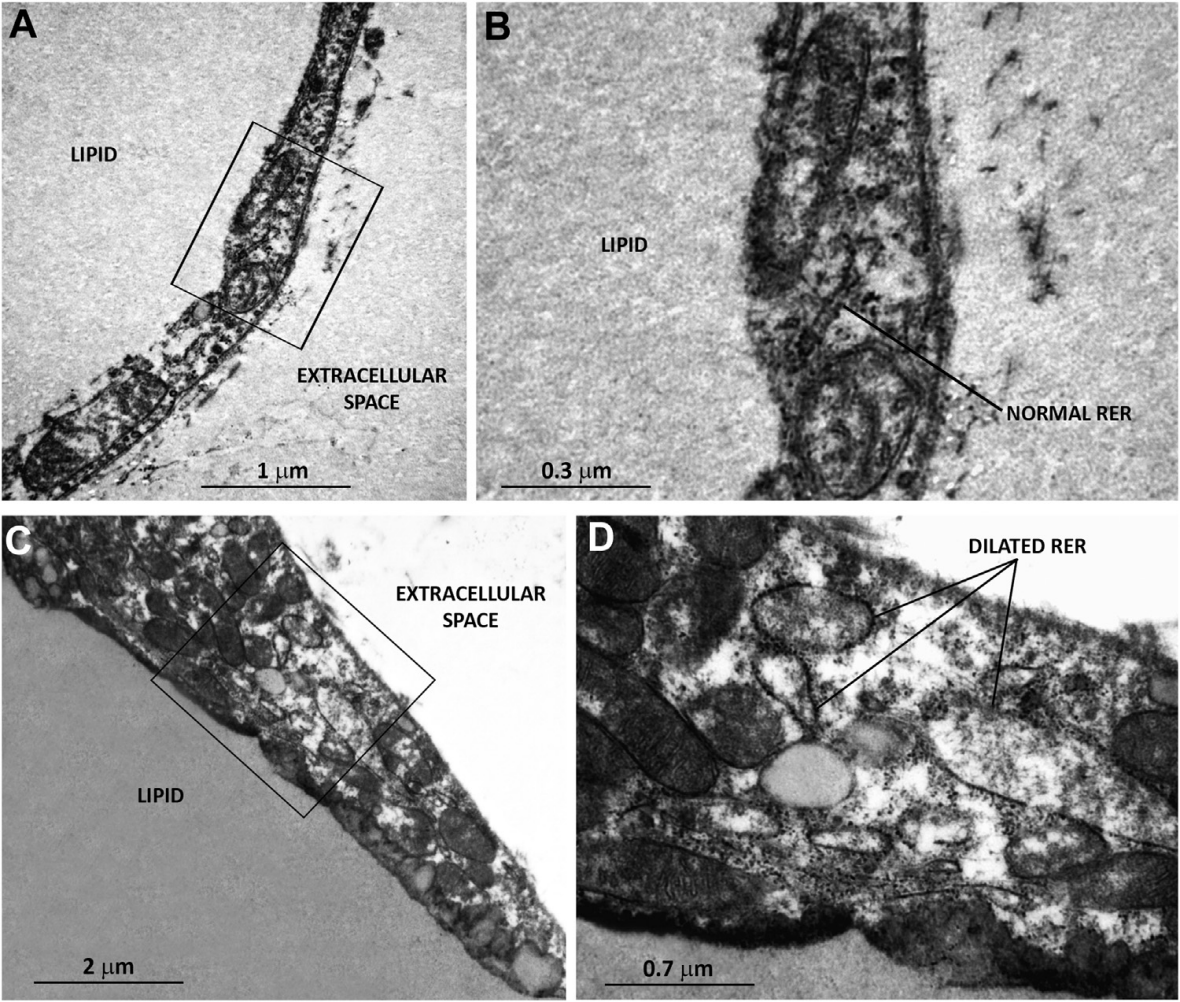
Transmission electron microscopy of eWAT from wild-type and *Hsl* knockout mice. White adipocytes from wild-type mice (A, B) exhibit normal ER structures, whereas adipocytes from *Hsl* knockout mice (C, D) contain dilated ER structures. B and D are enlargements of the corresponding areas framed in A and C, respectively.

**Fig. 3:**
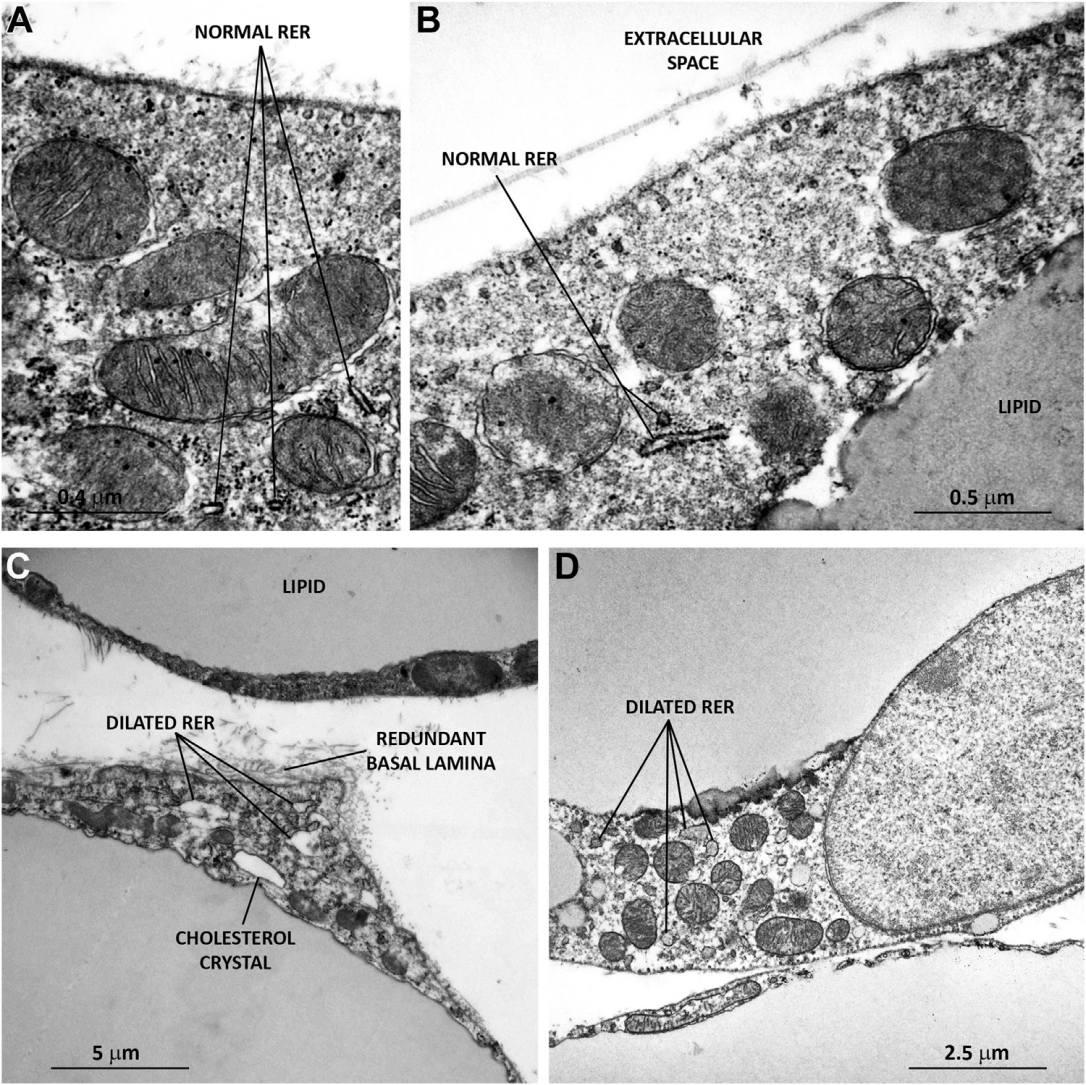
Transmission electron microscopy of iWAT from wild-type and *Hsl* knockout mice. In wild-type mice (A, B), white adipocytes contain normal rough ER structures. In *Hsl* knockout mice (C, D), adipocytes exhibit dilated rough ER structures and signs of cellular stress, including redundant basal lamina and cholesterol crystals.

Ultrastructural analyses of eWAT and iWAT confirmed the presence of dilated ER in the cytoplasm of numerous *Hsl* knockout adipocytes (Figs. 2 and 3). A visually evident dilated ER was found in about 50% of unilocular adipocytes in both depots. Moreover, unilocular adipocytes from *Hsl* knockout mice occasionally showed other features of stress, including the presence of cholesterol crystals in their cytoplasm and collagen accumulation close to the plasma membrane. Overall, molecular and morphological data demonstrated that HSL loss led to ER stress in both investigated WAT depots.

### HSL loss promotes inflammation in eWAT and reduced PPARγ signaling in eWAT and iWAT

We analyzed macrophage infiltration, inflammatory marker gene expression, and *Pparγ* expression in eWAT and iWAT of wild-type and *Hsl* knockout mice in order to study whether ER stress is linked to adipose tissue inflammation and dysfunction.

**Fig. 4:**
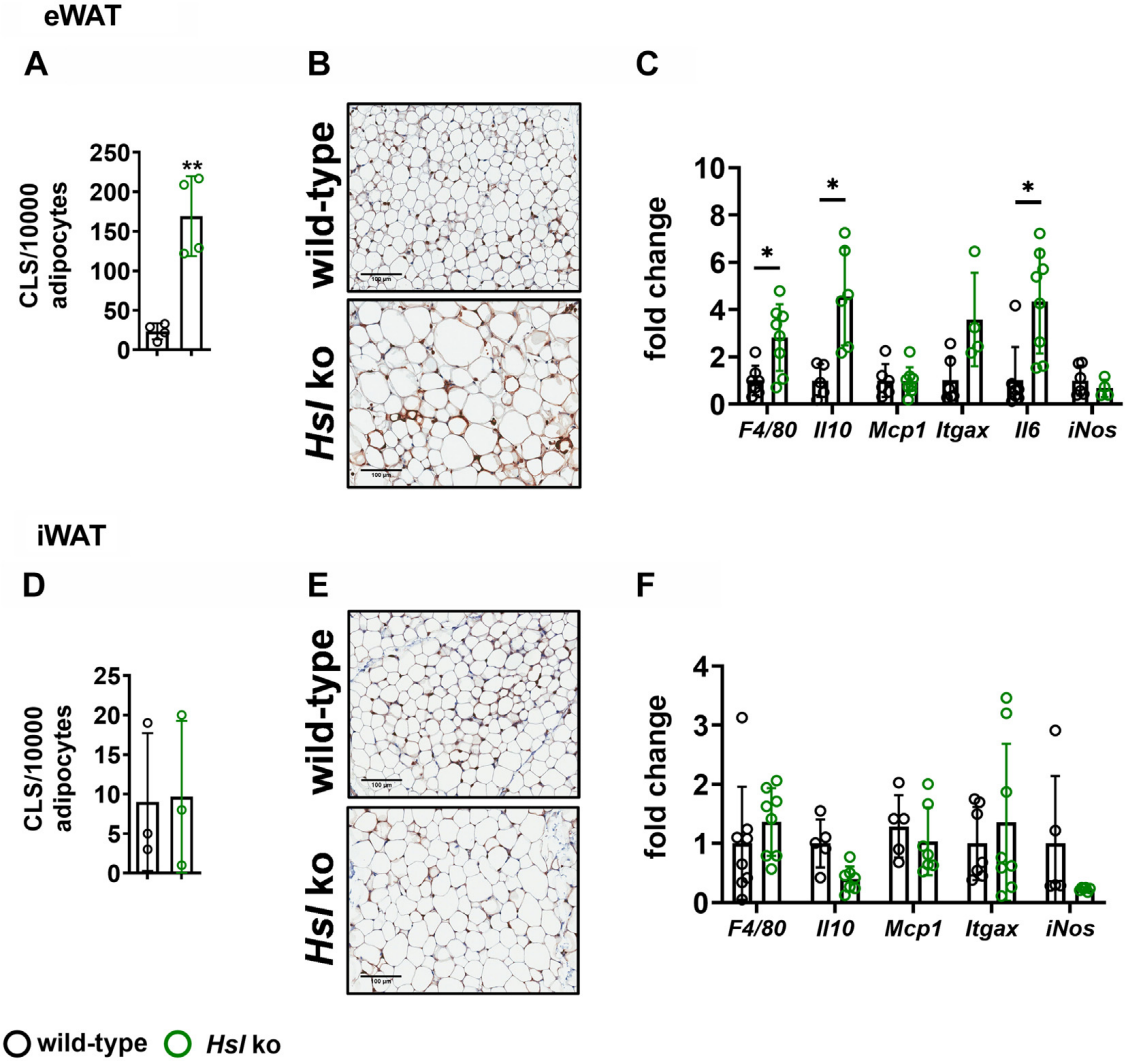
CLS density and inflammatory marker gene expression in eWAT and iWAT of wild-type and *Hsl* knockout mice. A: Quantification of CLS. B: Representative pictures of anti-MAC2 staining of eWAT (*n* = 4). C: mRNA expression of *F4/80*, *Il10*, *Mcp1*, *Itgax/Cd11c*, interleukin 6 (*Il6*), and inducible nitric oxide synthase (*iNos*) in eWAT of HSL knockout and wild-type mice (*n* = 4–8). D: Quantification of CLS (*n* = 3). E: Representative pictures of anti-MAC2 staining of iWAT. F: mRNA expression of *F4/80,Il10*, *Mcp1*, *Itgax/Cd11c*, *Il6*, and *iNos* in iWAT of *Hsl* knockout and wild-type mice (*n* = 4–8). The mRNA expression of inflammatory markers was measured by quantitative real-time PCR. Target gene abundance was normalized to *36b4* and/or hypoxanthine-guanine-phosphoribosyltransferase (*Hprt*) and expressed relative to wild-type levels of each marker. Data are represented as mean ± SD. Statistical significance was determined using unpaired two-tailed Student’s *t*-test, corrected for multiple comparisons using the Holm-Sidak method. (∗) *P* < 0.05 and (∗∗) *P* < 0.01.

**Fig. 5:**
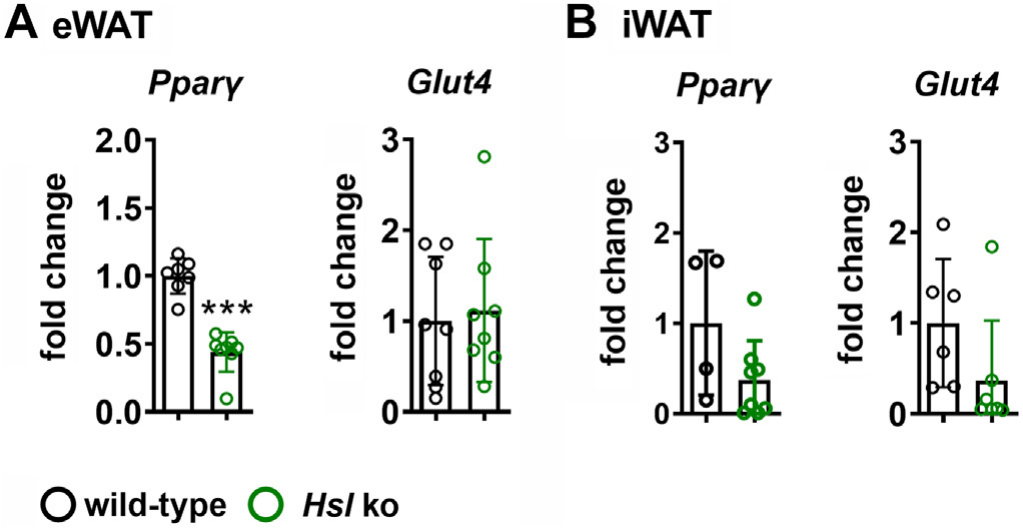
*Pparγ* und *Glut4* gene expression in eWAT and iWAT of wild-type and *Hsl* knockout mice. A: mRNA expression of *Pparγ* and *Glut4* in eWAT of *Hsl* knockout and wild-type mice (*n* = 7–8). B: mRNA expression of *Pparγ* and *Glut4* in iWAT of *Hsl* knockout and wild-type mice (*n* = 4–8). The mRNA expression was measured by quantitative real-time PCR. Target gene abundance was normalized to *36b4* and/or hypoxanthine-guanine-phosphoribosyltransferase (*Hprt*) and expressed relative to wild-type levels of each marker. Data are represented as mean ± SD. Statistical significance was determined using unpaired two-tailed Student’s *t*-test. (∗∗∗) *P* < 0.001.

eWAT of *Hsl* knockout mice showed adipocyte hypertrophy and an increased number of CLS and increased expression of the macrophage marker *F4/80* and *Itgax/CD11c* (Fig. 4A–C). Expression of inflammatory factors such as interleukin 6 (*Il6*) and interleukin 10 (*Il10*) was also significantly increased, whereas monocyte-chemoattractant protein 1 (*Mcp1*) and inducible nitric oxide synthase (*iNos*) remained unchanged (Fig. 4C). Although iWAT also showed upregulation of ER stress markers, no signs of tissue inflammation were observed, and CLS count and inflammatory marker gene expression was comparable among genotypes (Fig. 4D–F). Adipose tissue function was decreased in eWAT of *Hsl* knockout mice, reflected by decreased *Pparγ* expression but unchanged *Glut4* expression (Fig. 5A). In iWAT, *Pparγ* and *Glut4* expression were slightly but not significantly decreased (Fig. 5B). Despite pronounced eWAT inflammation and dysfunction, overall glucose tolerance and insulin sensitivity were not altered in *Hsl* knockout mice (supplemental Fig. S1A, B). However, protein kinase B (AKT) phosphorylation in saline-injected *Hsl* knockout animals was slightly but not significantly decreased in both eWAT and iWAT (supplemental Fig. S1C, D). Upon insulin stimulation, eWAT of *Hsl* knockout animals was less responsive and showed less AKT phosphorylation than eWAT from wild-type controls. In iWAT of *Hsl* knockout mice, there was only a slight but not significant reduction of AKT phosphorylation detectable after insulin injection.

### PPARγ activation attenuates inflammation but not ER stress in eWAT

We hypothesized that decreased PPARγ signaling in eWAT and iWAT might be causal for ER stress and inflammation. To investigate whether ER stress and inflammation are ameliorated in eWAT and iWAT, we fed wild-type mice and *Hsl* knockout a rosiglitazone-enriched diet. eWAT and iWAT of the rosiglitazone-treated wild-type and *Hsl* knockout mice were analyzed in terms of ER stress marker distribution and adipose tissue inflammation and compared with untreated wild-type and *Hsl* knockout mice.

**Fig. 6:**
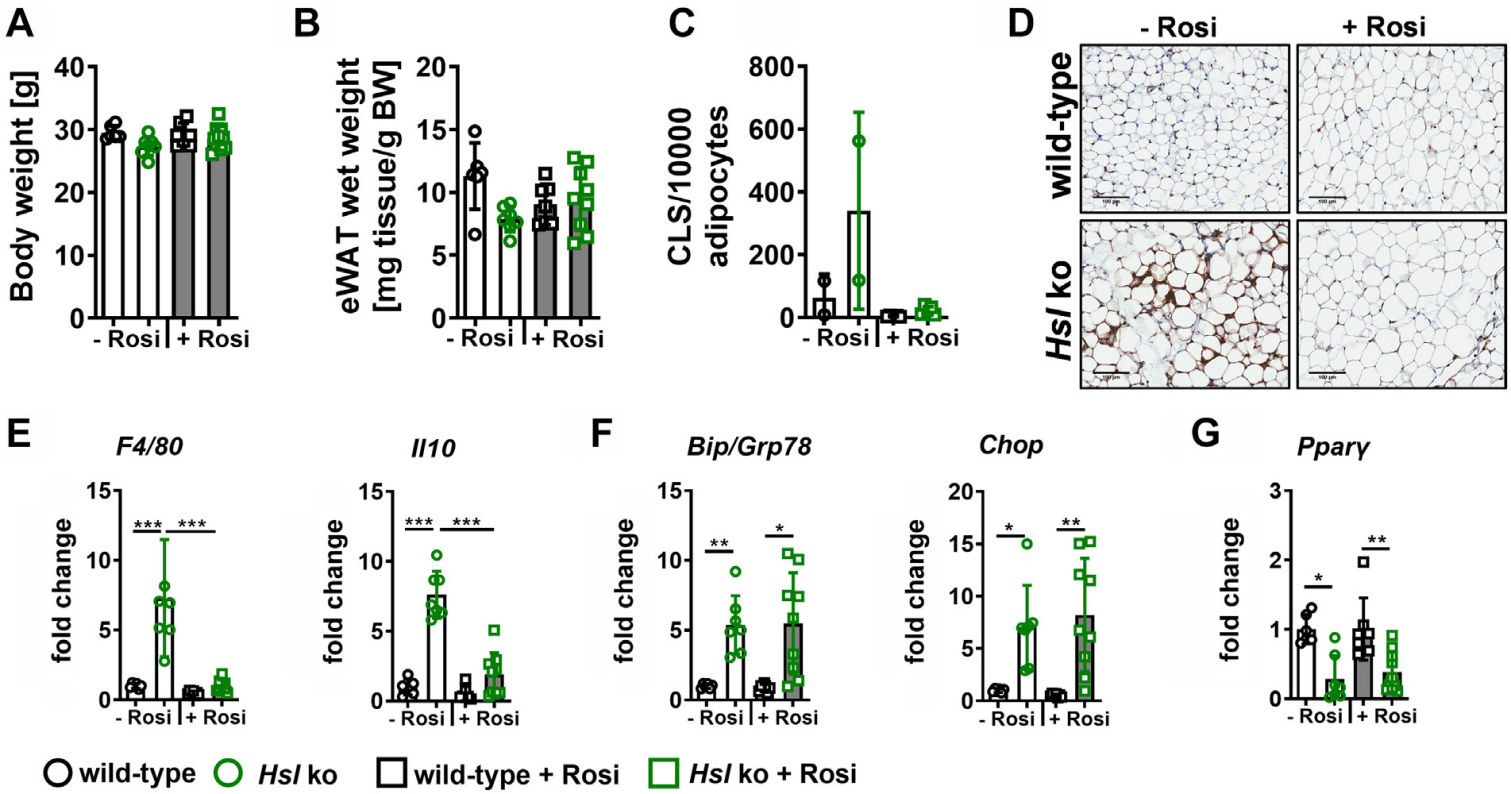
Body weight, inflammatory marker, ER stress marker, and *Pparγ* expression in eWAT after rosiglitazone treatment of *Hsl* knockout mice. A: Body weight and (B) eWAT wet weight of wild-type and *Hsl* knockout mice without (−Rosi) and with rosiglitazone treatment (+Rosi) (*n* = 6–9). C: CLS count in eWAT of wild-type and *Hsl* knockout mice without (−Rosi) and with rosiglitazone treatment (+Rosi) (*n* = 2–5). D: Representative pictures of anti-MAC2 staining of eWAT of wild-type and *Hsl* knockout mice without (−Rosi) and with rosiglitazone treatment (+Rosi). E: mRNA expression of inflammatory markers (*F4/80*, *Il10*) of wild-type and *Hsl* knockout mice without (−Rosi) and with rosiglitazone treatment (+Rosi). F: ER stress marker and (G) *Pparγ* mRNA expression of wild-type and *Hsl* knockout mice without (−Rosi) and with rosiglitazone treatment (+Rosi) (*n* = 6–9). The mRNA expression was measured by quantitative real-time PCR. Target gene abundance was normalized to *36b4* and hypoxanthine-guanine-phosphoribosyltransferase (*Hprt*) and expressed relative to wild-type levels of each marker. Data are given as mean ± SD. Statistical significance was determined using two-way ANOVA and Tukey multiple comparisons test. (∗) *P* < 0.05; (∗∗) *P* < 0.01; and (∗∗∗) *P* < 0.001.

Rosiglitazone treatment did not affect body weight in wild-type and *Hsl* knockout mice. eWAT mass was slightly decreased in untreated wild-type mice compared with untreated HSL knockout littermates (Fig. 6A, B). Mac-2 stainings and CLS quantification of eWAT sections showed that 20 days of rosiglitazone treatment diminished macrophage infiltration and CLS formation in eWAT of *Hsl* knockout mice (Fig. 6C, D) but still remained higher in *Hsl* knockout mice. In line with this, *F4/80* and *Il10* expression was significantly decreased after rosiglitazone treatment (Fig. 6E). Despite decreased inflammation, ER stress marker expression remained at similar levels when compared with wild-type mice (Fig. 6F). *Pparγ* expression, however, was not affected (Fig. 6G). In iWAT of rosiglitazone-treated *Hsl* knockout mice, rosiglitazone treatment had no effect on iWAT mass or inflammatory marker expression (supplemental Fig. S2A, B), but ER stress markers seemed to be even slightly increased upon rosiglitazone treatment (supplemental Fig. S2C). *Pparγ* expression was significantly increased in wild-type mice upon rosiglitazone treatment but only showed a slight increase in *Hsl* knockout mice (supplemental Fig. S2D). Gene expression of the *Pparγ* targets leptin, *Plin1*, and adiponectin (*AdipoQ*) were decreased in *Hsl* knockout mice compared with wild-type controls and showed a trend toward upregulation upon rosiglitazone treatment in eWAT and iWAT except for leptin (supplemental Fig. S3). In eWAT, *Plin1* was significantly upregulated in *Hsl* knockout mice upon rosiglitazone treatment (supplemental Fig. S3).

### Rosiglitazone treatment decreased postprandial increase in plasma FA and normalized leptin levels but did not affect DAG or Cer accumulation in eWAT of *Hsl* knockout mice

**Fig. 7:**
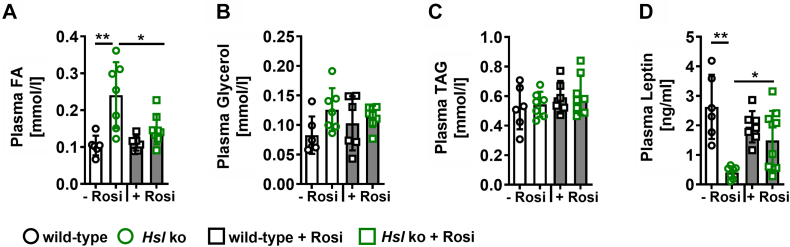
Plasma concentrations of FA, glycerol, TAG, and leptin in wild-type and *Hsl* knockout mice treated without or with rosiglitazone. A: Plasma FA, (B) plasma glycerol, (C) plasma TAG, and (D) plasma leptin levels of wild-type and *Hsl* knockout mice after 2 h of refeeding with (+Rosi) or without rosiglitazone (−Rosi) (*n* = 6–10). Data are represented as mean ± SD. Statistical significance was determined by two-way ANOVA and Tukey multiple comparisons test. (∗) *P* < 0.05 and (∗∗) *P* < 0.01.

Plasma FAs were significantly elevated in refed *Hsl* knockout mice. This effect was completely missing in *Hsl* knockout mice treated with rosiglitazone (Fig. 7A). No significant changes in plasma glycerol (Fig. 7B) and plasma TAG (Fig. 7C) were detected. In ad libitum-fed *Hsl* knockout mice, plasma FA and TAG levels remained unchanged but were significantly decreased upon rosiglitazone treatment in *Hsl* knockout mice (supplemental Fig. S4). Leptin levels were significantly decreased in *Hsl* knockout mice but reached wild-type levels upon rosiglitazone treatment (Fig. 7D), which indicates partially restored adipose tissue function.

**Fig. 8:**
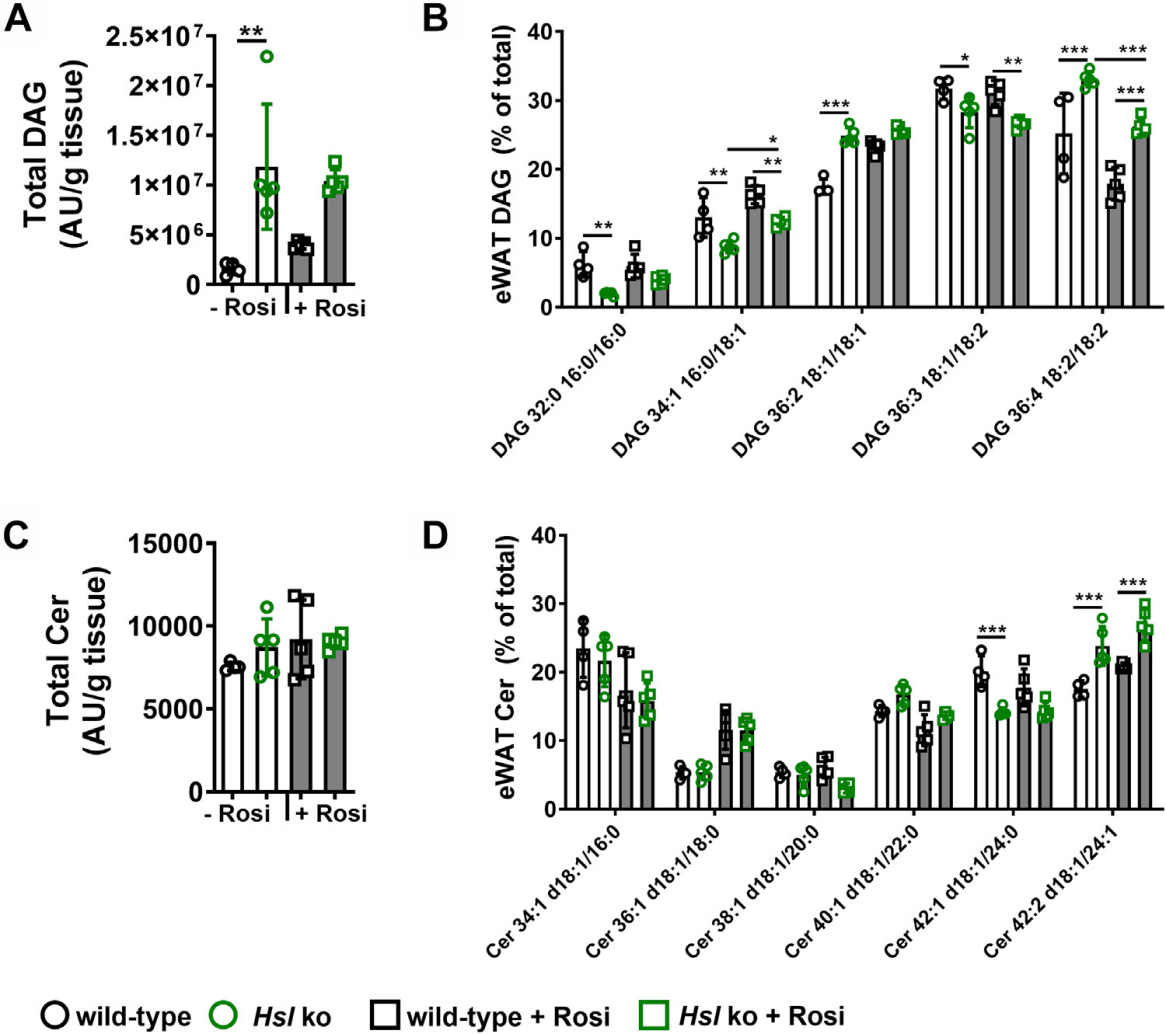
DAG and Cer levels in eWAT of untreated and rosiglitazone-treated wild-type and *Hsl* knockout mice. A: Total DAG levels of eWAT of wild-type and *Hsl* knockout mice without (−Rosi) and with rosiglitazone treatment (+Rosi) and (B) DAG species distribution given as percent of total DAG signal (*n* = 4–5). C: Total Cer levels of eWAT of wild-type and *Hsl* knockout mice without (−Rosi) and with rosiglitazone treatment (+Rosi), and (D) Cer species distribution given as percent of total Cer signal (*n* = 4–5). Data are represented as mean ± SD. Statistical significance was determined by two-way ANOVA and Tukey multiple comparisons test. (∗) *P* < 0.05; (∗∗) *P* < 0.01; and (∗∗∗) *P* < 0.001.

Since inflammation and ER stress were more pronounced in eWAT, we analyzed DAG and Cer levels of eWAT from wild-type and *Hsl* knockout mice with and without rosiglitazone treatment. We found that DAG accumulation was pronounced in eWAT of *Hsl* knockout mice. Rosiglitazone treatment did not affect the total DAG levels in eWAT of *Hsl* knockout mice but slightly increased total DAG in wild-type mice (Fig. 8A). No robust effect of rosiglitazone treatment on FA species distribution in the most abundant DAG species (Fig. 8B) in *Hsl* knockout mice was detected. Overall, significant differences in the DAG FA species distribution occurred among genotypes. DAG 32:0 (16:0/16:0) and DAG 34:1 (16:0/18:1) levels were significantly decreased in *Hsl* knockout compared with wild-type mice, whereas DAG 36:2 (18:1/18:1) and DAG 36:4 (18:2/18:2) levels were significantly increased (Fig. 8B). The differences among genotypes largely remained unaffected by rosiglitazone treatment. Only DAG 36:4 (18:2/18:2) levels were increased in eWAT of *Hsl* knockout mice and reduced to wild-type mice levels after rosiglitazone treatment (Fig. 8B). DAG 34:1 (16:0/18:1) levels were significantly decreased in *Hsl* knockout mice but slightly increased with rosiglitazone treatment, whereas wild-type levels remained unaffected by the treatment. Total eWAT Cer levels did not differ with respect to genotypes and treatment (Fig. 8C). Similar to DAG FA species distribution, also Cer FA species distribution either showed only a treatment effect, where rosiglitazone treatment only altered Cer levels without any difference among genotypes. Cer 34:1 (d18:1/16:0) and Cer 40:1 (d18:1/22:0) levels were slightly decreased by rosiglitazone treatment, and Cer 36:1 (d18:1/18:0) levels were increased, but almost no changes among genotypes occurred. Cer 42:1 (d18:1/24:0) levels were significantly decreased in *Hsl* knockout mice but not affected by rosiglitazone treatment. Cer 42:2 (d18:1/24:1) levels were increased in untreated *Hsl* knockout mice and only showed a slight trend of higher levels after rosiglitazone treatment (Fig. 8D).

## Discussion

Data from the present study showed that ER stress occurred to the same extent in both eWAT and iWAT of *Hsl* knockout mice. We observed macrophage infiltration and increased inflammatory marker expression in eWAT, where rosiglitazone treatment attenuated inflammation but not ER stress. Rosiglitazone treatment did not affect DAG or Cer deposition in eWAT of *Hsl* knockout mice.

The expression of ER stress markers and the ultrastructural analyses of eWAT and iWAT demonstrated pronounced ER stress in both investigated WAT depots of *Hsl* knockout mice. Previous work from Pinent *et al.* (24) and Mottillo *et al.* (25) has indicated that altered HSL activity might interfere with ER stress response. They have shown that loss of HSL or ATGL differentially affects transcription of several metabolic genes in various tissues such as WAT, heart, or liver tissue (24). Microarray analysis of WAT and further gene ontology annotation has shown that ER stress response was upregulated in WAT of *Hsl* knockout mice (24), but the underlying mechanisms have not been investigated. Mottillo *et al.* have suggested that inflammatory response and ER stress in adipose tissue in vivo and in adipocytes in vitro are promoted by fully activated lipolysis. The selective HSL inhibitor suppressed activated lipolysis and was in turn suppressing inflammation but not ER stress. HSL inhibition rather tended to further increase ER stress than inhibiting it (25). In contrast to Mottillo *et al.*, chronic loss of HSL activity as it occurs in *Hsl* knockout mice did not prevent but induced inflammation in WAT (10, 11).

Our and previous work showed that *Hsl* knockout mice exhibit adipose tissue inflammation (10, 11). However, we found that inflammation mainly occurred in the visceral adipose tissue depot eWAT and did not occur in iWAT under the present conditions. Previous data from our group also indicated that eWAT inflammation is not associated to systemic inflammation, since inflammatory cytokines were not increased in plasma of *Hsl* knockout mice (supplemental Fig. S5). Previous studies from Cinti *et al.* (10) and Hansson *et al.* (11) have also observed inflammation in visceral adipose tissue, but we also investigated subcutaneous adipose tissue. The data from our study revealed that ER stress occurred to the same extent in eWAT and iWAT, but macrophage infiltration and inflammation were only present in eWAT. This supports the concept that pronounced ER stress does not cause initiation of adipose tissue inflammation, at least in WAT. A previous study from our group showed that *Atgl* knockout mice have massive inflammation in brown adipose tissue without any signs of ER stress, indicating that adipose tissue inflammation and ER stress are not consequently linked (33).

*Pparγ* and respective target gene expression was reduced in eWAT and iWAT indicating reduced adipose tissue functionality in both WAT depots. Treatment of *Hsl* knockout mice with the PPARγ agonist rosiglitazone diminished inflammation in eWAT of *Hsl* knockout mice but did not resolve ER stress. Rosiglitazone treatment also partially restored expression of PPARγ target genes in eWAT and iWAT (supplemental Fig. S5) and normalized plasma leptin levels. Shen *et al.* (6) have shown that FAs cleaved by HSL are needed for PPARγ activation. In line with these observations, we found lowered eWAT mass in *Hsl* knockout mice, which was partially restored upon rosiglitazone treatment. Interestingly, our data indicate that adipocyte loss and inflammation predominantly occurs in eWAT, where PPARγ signaling seemed to be decreased to a greater extent in eWAT than in iWAT. In high-fat diet-fed adipocyte-specific *Hsl* knockout mice however, eWAT and iWAT inflammation occurred at the same time, but it seems that inflammation was much more pronounced in eWAT, whereas PPARγ signaling was decreased in both depots (34). We speculate that upon long-term high-fat diet challenge, both depots are similarly affected.

Although we and others found significant DAG accumulation in adipose tissue of *Hsl* knockout mice, our results did not identify DAG or Cer lipotoxicity as source of inflammation. We found that neither DAG levels nor Cer levels in eWAT were altered by rosiglitazone treatment. This is in line with results from Shen *et al.* (6) who did not detect any changes in DAG levels upon rosiglitazone treatment of *Hsl* knockout mice. We found a shifted distribution of DAG species, with DAG 18:2/18:2 levels elevated in untreated *Hsl* knockout mice, which was reversed upon rosiglitazone treatment. There is evidence that DAG and Cer accumulation in general and also accumulation of some DAG species like DAG 18:2/18:2 contribute to the development of insulin resistance in muscle in vivo (35). However, if and how DAGs contribute to insulin resistance is still a matter of debate, since other studies have found no association of increased tissue DAG levels with insulin resistance (36). Increased DAG levels in macrophages have even suppressed an inflammatory burst and might be important in preventing sepsis in vivo (37). DAG and Cer concentrations are not or only partially responsible for ER stress and adipose tissue inflammation in *Hsl* knockout mice according to our data. However, since rosiglitazone treatment did not ameliorate ER stress and did not reduce DAG accumulation, we cannot exclude that DAG accumulation might drive ER stress. Previous studies have shown that ER stress is driven by DAG accumulation in vitro in cardiomyocytes (18). In line with our findings, pioglitazone treatment in humans did not affect overall Cer levels in adipose tissue. Although sphingosine (d16:0) and (d18:1) containing ceramides have been previously described for both eWAT and iWAT (38), we primarily focused on sphingosine (d18:1) containing ceramides. Multiple studies were able to show that especially Cer d18:1 species were associated to insulin resistance in humans (39, 40, 41), and Cer d18:1/16:0 and Cer d18:1/18:0 were specifically more abundant in eWAT of high-fat diet-fed mice (38). We found that rosiglitazone treatment decreased postprandial plasma FA concentration in *Hsl* knockout mice, which could be associated with reduction of adipose tissue inflammation. A previous study from Zimmermann (5) *et al.* has shown that decreased PPARγ signaling impaired FA re-esterification in WAT and thereby compensated the lipolytic defect in *Hsl* knockout mice. Moreover, they have reported elevated palmitate levels in adipose tissue (3). Increased palmitate levels affected by lowered PPARγ signaling could contribute to adipose tissue inflammation by inducing inflammatory signaling (42). Although an overload in FAs, especially palmitate, has been associated with the development of ER stress (43), decreased postprandial plasma FA concentration in *Hsl* knockout mice did not affect ER stress markers in eWAT and iWAT. Recently, Chitraju *et al.* (44) have demonstrated that diacylglycerol-O-acyltransferase-1-mediated FA re-esterification is crucial to prevent FA-induced ER stress during maximal lipolysis in adipocytes. Loss of diacylglycerol-O-acyltransferase-1, specifically in adipocytes, induced ER stress and inflammation in WAT by suppressing re-esterification and FA detoxification. ER stress was not suppressed in our study, although we found lowered postprandial plasma FA concentrations, and therefore most likely efficient re-esterification in rosiglitazone-treated mice. Since our study was carried out under postprandial conditions when lipolysis is suppressed, we speculate that ER stress is induced by different mechanisms during maximal lipolysis or postprandial.

Lipolysis-derived FAs are crucial for PPARγ activation (6); a shift in lipolytic activity in mice and humans might affect adipogenesis and adipose tissue functionality in vivo. A study in humans demonstrated that female obesity alters lipolytic activity in subcutaneous adipose tissue, which goes hand in hand with diminished functionality, impaired glucose metabolism, and weight gain (45). Data obtained from an Amish population with various mutants of HSL showed that the HSL loss in humans leads to impaired glucose metabolism (8), a status that is not fully supported by our data in mice. Furthermore, impaired HSL function decreases subcutaneous adipose tissue mass in humans (8), whereas in our study, primarily visceral adipose tissue mass declined in mice. But impaired HSL activity has also been associated with inflammation and decreased expression of PPARγ target genes in subcutaneous adipose tissue (8). Data from our studies on *Hsl* knockout mice and also data from studies on human HSL functionality (8, 45) demonstrate that HSL is indispensable for adipose tissue function and that a shift in HSL activity, for example in obesity, causes impaired adipose tissue function and inflammation and is possibly responsible for the development of obesity-related disorders. HSL seems to have a certain depot specificity that appears to be different in mice and humans.

## Conclusion

HSL loss promoted ER stress in both visceral (eWAT) and subcutaneous adipose tissue (iWAT) to the same extent. Inflammation and macrophage infiltration were only detected in eWAT and PPARγ activation attenuated inflammation but not ER stress in eWAT. Rosiglitazone treatment did not affect the DAG or Cer accumulation in eWAT of *Hsl* knockout mice, but it altered plasma FA concentration. We assume that reduced FA re-esterification in *Hsl* knockout mice promotes inflammatory signaling in eWAT, which is reversed by rosiglitazone treatment. Further studies on HSL depot-specificity in mice and humans are necessary to fully understand the role of HSL in obesity and associated comorbidities.

## Data Availability

The datasets generated and analyzed during the current study are available from the corresponding author on reasonable request. All data generated and analyzed during this study are included in this published article (and its supplemental data files).

## Supplemental Data

This article contains supplemental data.

## Conflict of Interest

The authors declare that they have no conflicts of interest with the contents of this article.
