# Supplementary material for: Rosiglitazone Reverses Inflammation in Epididymal White Adipose Tissue in Hormone-Sensitive Lipase-Knockout Mice
Source: J Lipid Res. 2022 Oct 20;64(1):100305. doi: 10.1016/j.jlr.2022.100305 (PMC9760656; doi:10.1016/j.jlr.2022.100305)
Supplement: Supplementary Material [file mmc1.docx]

SUPPLEMENTARY MATERIAL

METHODS

***Glucose tolerance test (GTT) and insulin tolerance test (ITT)***

Glucose tolerance was monitored in awake mice that were fasted for 6 h and insulin tolerance was monitored in awake mice that were fasted for 4 h. For GTT, mice received an intraperitoneal injection of 1.5 g glucose per kilogram body weight. Blood was taken by tail vein puncture, and glucose levels were determined at the indicated time points using the Abbott FreeStyle lite glucometer (Abbott, USA). For ITT, mice received an intraperitoneal injection of 0.5 IU insulin per kilogram of body weight.

***Tissue specific insulin sensitivity test (IST)***

To analyze insulin sensitivity/insulin signaling in eWAT and iWAT of wild-type and *Hsl* knock-out mice, mice were fasted overnight (12 h) and injected with 0.75 IU/kg (Dilution 0.2 IU/ml) insulin or saline control and sacrificed 10 min after injection. eWAT and iWAT were excised and snap frozen for immunoblot analysis. 30 mg tissue was homogenized on ice in RIPA buffer supplemented with 20 µg ml^−1^ leupeptin, 2 µg ml^−1^ antipain, and 1 µg ml^−1^ pepstatin and PhosSTOP (Merck, Germany) and incubated for 1h on ice. Then, samples were centrifuged for 30 min at 4°C and 20.000 g and supernatant was collected using a syringe (excluding floating fatty layer). 15 µg protein was used for immunoblot analysis. Phosphorylation of AKT (pAKT) was analysed by using a primary antibody specific to the Ser473 phosphorylation site, pAKT (Ser473) (1:1000, # 9271, Cell Signaling Technology, MA, USA). Total AKT was assayed with AKT primary antibody (1:1000, # C67E7, Cell Signaling Technology, Boston, USA). Proteins were visualized using Clarity™ substrate and the ChemiDoc™system (Bio-Rad Laboratories GmbH, Germany). Signal density was determined with the ChemiDoc™system software (Bio-Rad Laboratories GmbH, Germany).

***ELISA and plasma parameters***

Blood samples were collected by retro-orbital puncture from ISOflo® (Abbott, Abbott Park, IL, USA) anesthetized 8-10 week old male wild-type or *Hsl* knock-out mice either fed or overnight fasted. Clotted blood samples were centrifuged 15 min at 4°C at 3600 rpm. Plasma was transferred to a fresh test tube and stored until use at -80°C. Repeated freeze and thaw cycles were avoided. ELISAs were performed according to manufacturer’s instructions. Plasma IL10 was analyzed by using the mouse IL-10 Quantikine kit (#M1000, R&D Systems, Inc, MN, USA) and MCP1 by using the mouse CCL2/JE/MCP-1 Quantikine kit (#MJE00, R&D Systems, Inc, MN, USA).

RESULTS**Supplementary Figure 1: GTT, ITT and tissue specific insulin signaling of wild-type and *Hsl* knock-out mice.** A) GTT and B) ITT of wild-type and *Hsl* knock-out mice (n = 5-3). C) Representative immunoblot of pAKT and total AKT and quantification of pAKT and AKT ratio in eWAT of wild-type and *Hsl* knock-out mice (n = 3). D) Representative immunoblot of pAKT and total AKT and quantification of pAKT and AKT ratio in iWAT of wild-type and *Hsl* knock-out mice (n = 3-4). Data are presented as mean ± SD. Statistical significance for the GTT and ITT was determined by two-way ANOVA and Bonferroni post-hoc test and for insulin signaling determined by two-way ANOVA and Tukey’s post-hoc test.


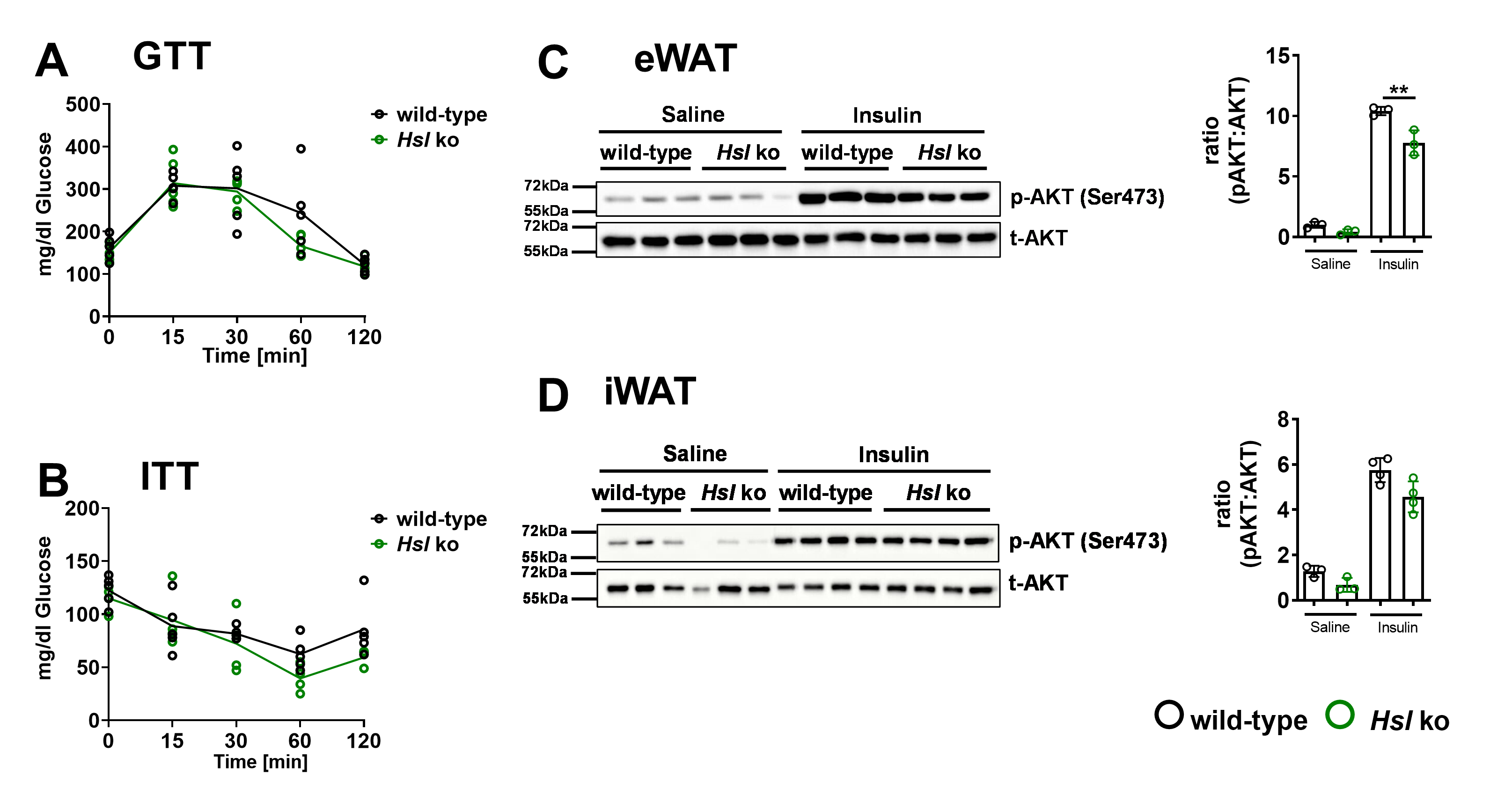

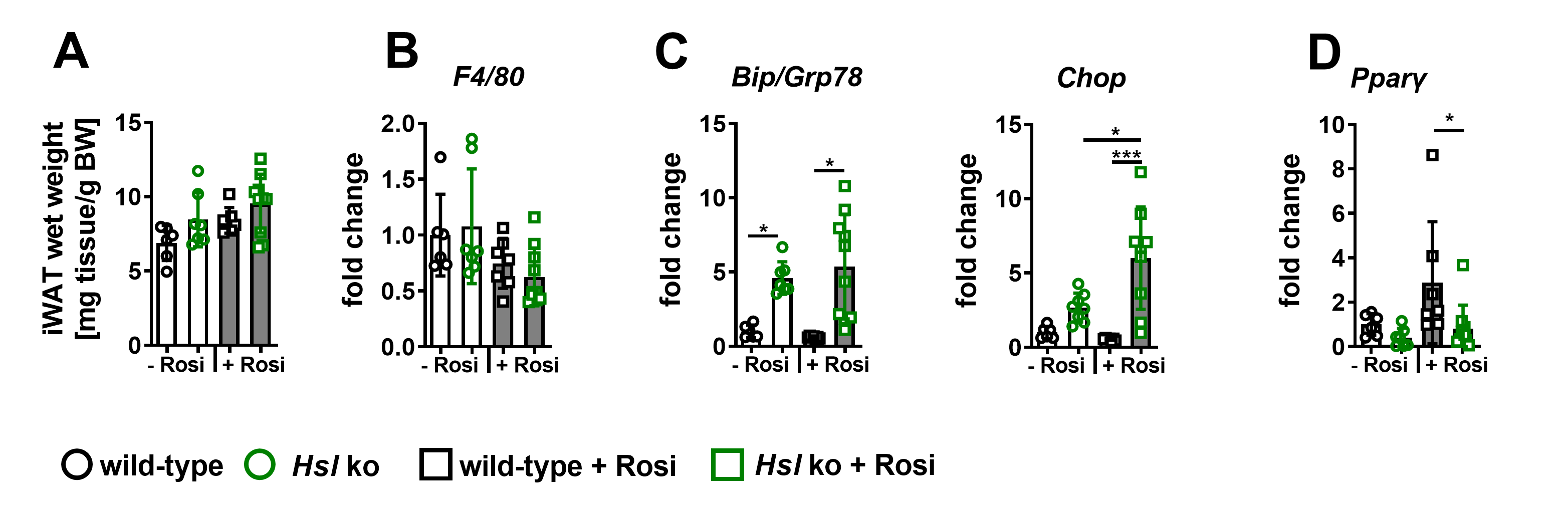


**Supplementary Figure 2: Inflammatory markers, ER stress markers and *Pparγ* in iWAT of rosiglitazone untreated and treated wild-type and *Hsl* knock-out mice.**. A) iWAT wet weight of *Hsl* knock-out mice and wild-type mice without (-Rosi) and with rosiglitazone treatment (+ Rosi). B) mRNA expression of the inflammatory markers *F4/80*, the ER stress markers C) *Bip*/*Grp78* and *Chop* and D) *Pparγ* of *Hsl* knock-out mice and wild-type mice without (-Rosi) and with rosiglitazone treatment (+ Rosi) (n = 6-10). Relative gene expression was measured by quantitative real-time PCR. Target gene abundance was normalized to *36b4* and *Hprt* and expressed relative to wild-type levels of each marker. Data are presented as mean ± SD. Statistical significance was determined by two-way ANOVA and Tukey’s post-hoc test (*) p < 0.05; (***) p < 0.001.


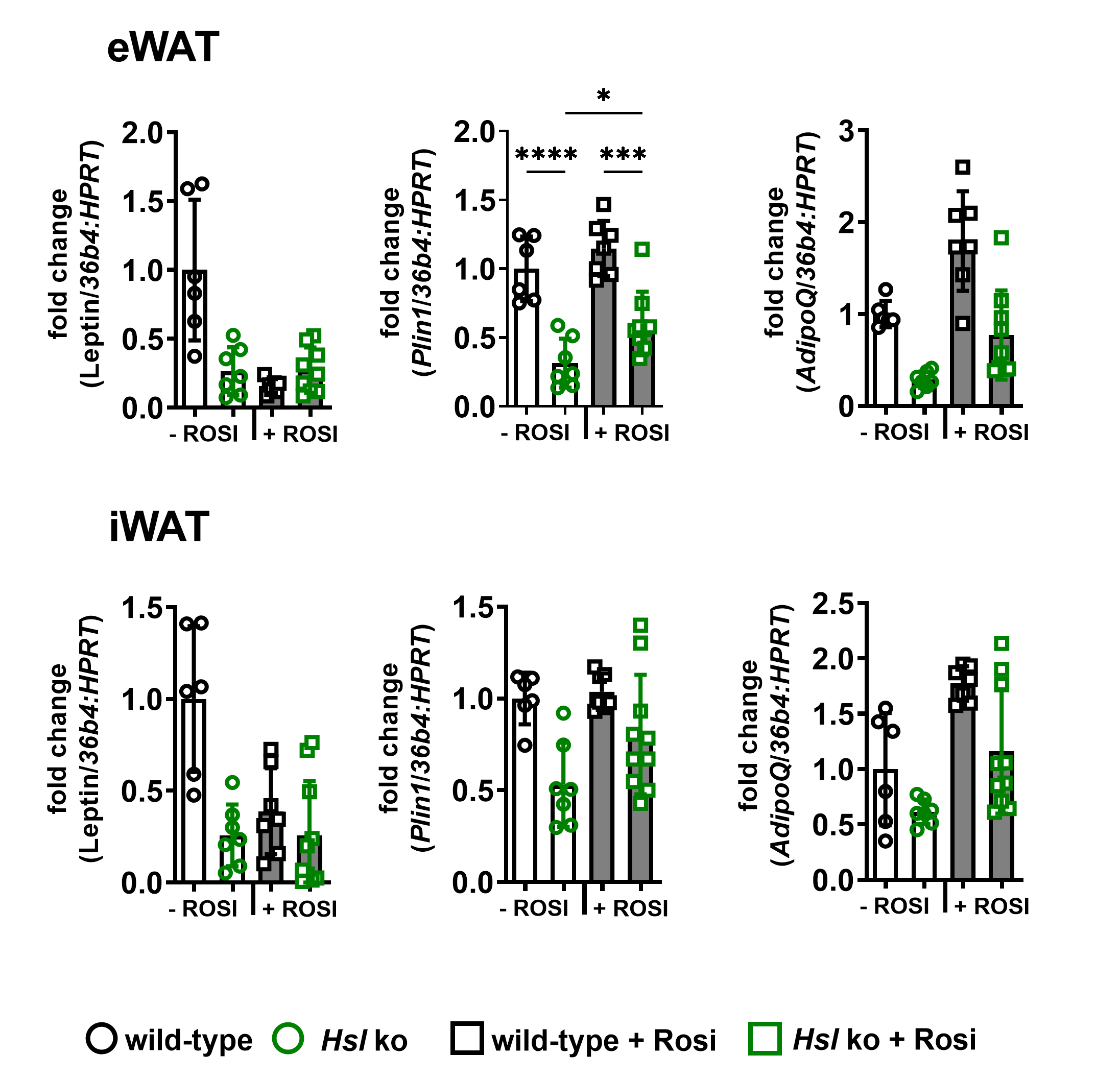


**Supplementary Figure 3: Leptin, perilipin (*Plin1*) and adiponectin (*AdipoQ*) expression in eWAT and iWAT of rosiglitazone untreated and treated wild-type and *Hsl* knock-out mice.** Gene expression of the *Pparγ* target genes leptin, Plin1 and AdipoQ were measured in eWAT and iWAT of *Hsl* knock-out mice and wild-type mice without (-Rosi) and with rosiglitazone treatment (+ Rosi) (n = 6-10). Relative gene expression was measured by quantitative real-time PCR. Target gene abundance was normalized to *36b4* and *Hprt* and expressed relative to wild-type levels of each marker. Data are presented as mean ± SD. Statistical significance was determined by two-way ANOVA and Tukey’s post-hoc test (*) p < 0.05; (***) p < 0.001, (****) p < 0.0001.


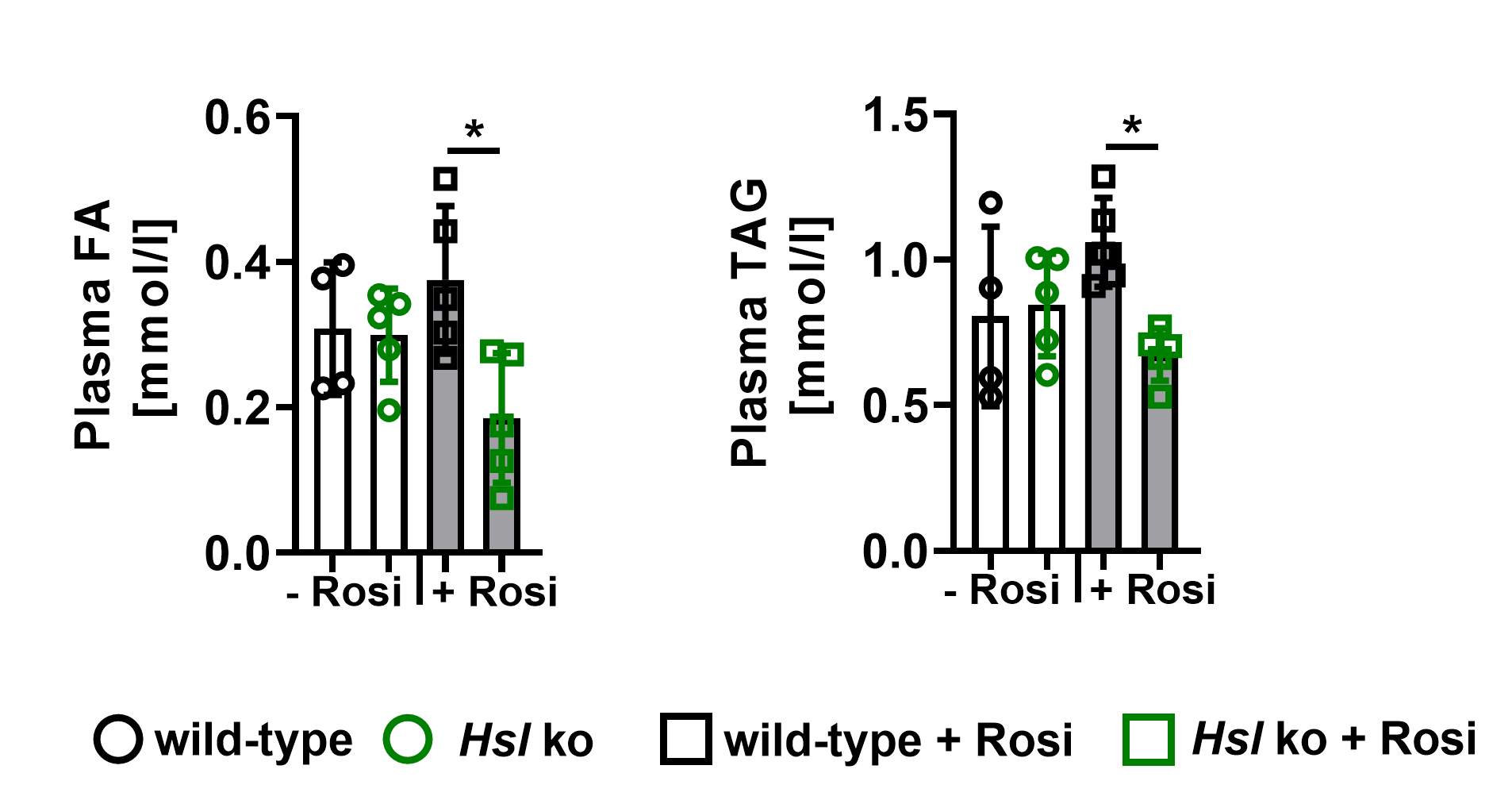


**Supplementary Figure 4: Plasma FA and triacylglycerol (TAG) concentrations in ad libitum fed wild-type and *Hsl* knock-out mice treated without or with rosiglitazone**. Data are represented as mean ± SD. Statistical significance was determined by two-way ANOVA and Tukey’s multiple comparisons test (*) p < 0.05. (n = 4-5)


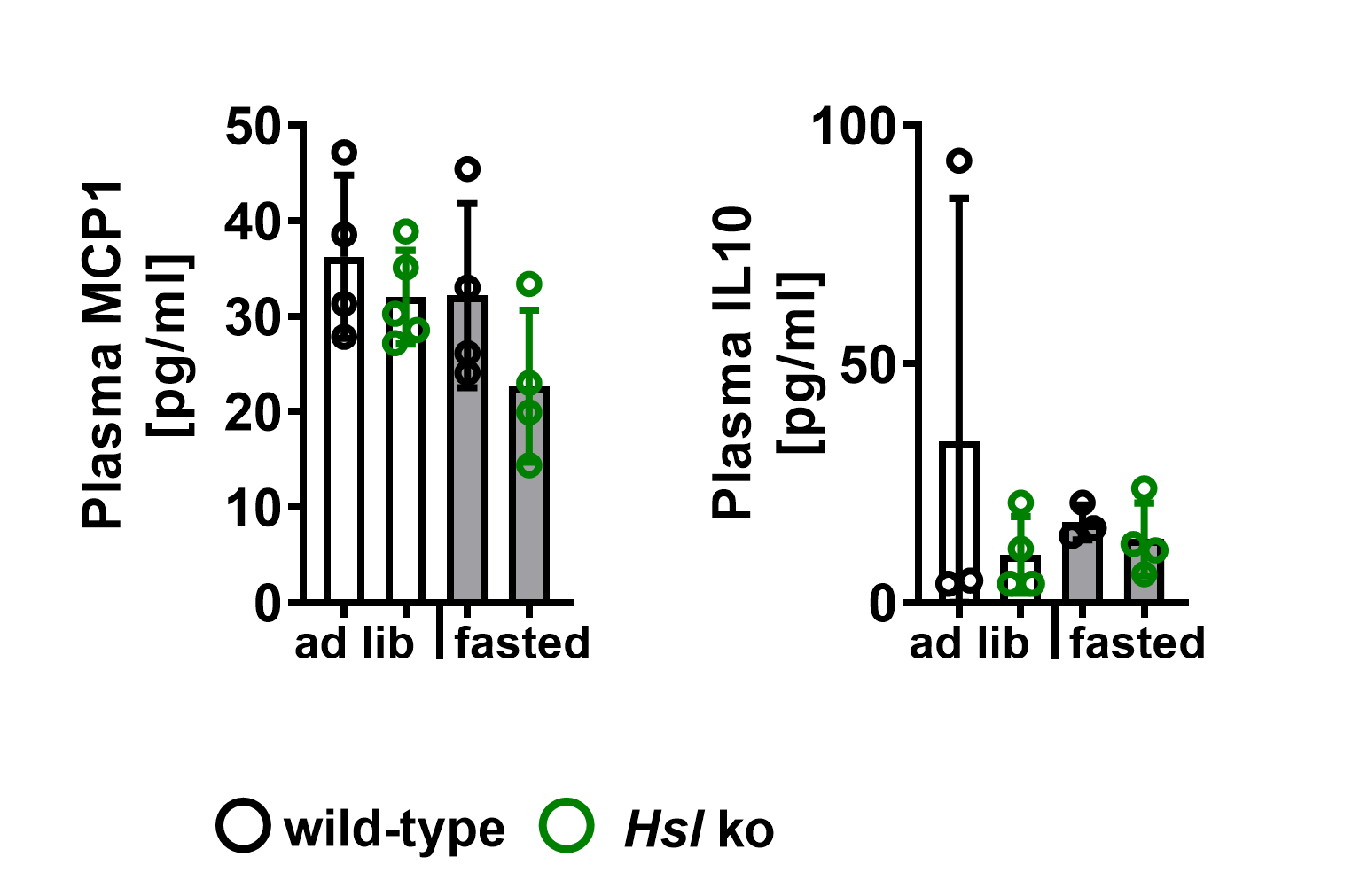


**Supplementary Figure 5: Plasma concentrations of MCP1 and IL10 in wild-type and *Hsl* knock-out mice**. Plasma MCP1 and IL10 levels of wild-type and *Hsl* knock-out mice in ad libitum fed and overnight fasted mice (n = 3-5). Data are represented as mean ± SD. Statistical significance was determined by two-way ANOVA and Tukey’s multiple comparisons test.


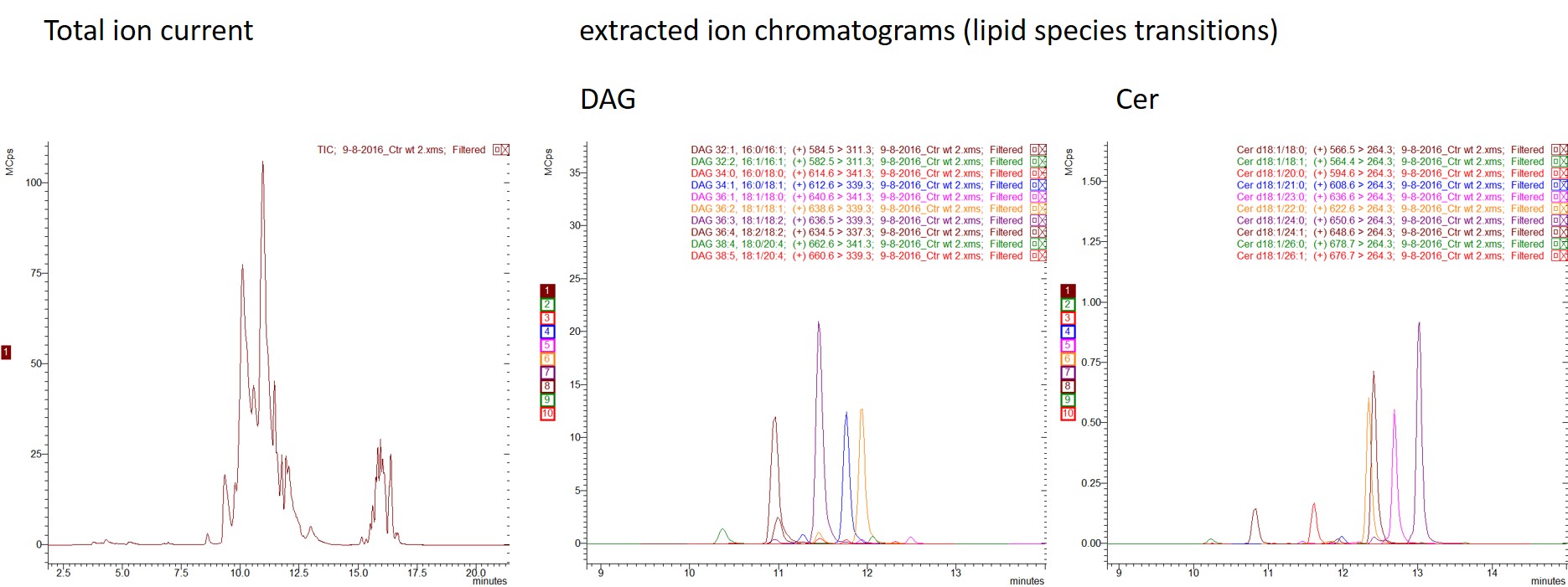


**Supplementary Figure 6: Total ion current and extracted ion chromatograms used in DAG and Cer analysis.**
